# Observational long-term follow-up study of rapid food oral immunotherapy with omalizumab

**DOI:** 10.1186/s13223-017-0223-8

**Published:** 2017-12-21

**Authors:** Sandra Andorf, Monali Manohar, Tina Dominguez, Whitney Block, Dana Tupa, Rohun A. Kshirsagar, Vanitha Sampath, R. Sharon Chinthrajah, Kari C. Nadeau

**Affiliations:** Sean N. Parker Center for Allergy and Asthma Research at Stanford University, 269 Campus Drive CCSR 3215, MC 5366, Stanford, CA 94305-5101 USA

**Keywords:** Follow-up, Food allergy, Maintenance dosing, Omalizumab, Oral immunotherapy

## Abstract

**Background:**

A number of clinical studies focused on treating a single food allergy through oral immunotherapy (OIT) with adjunctive omalizumab treatment have been published. We previously demonstrated safety and tolerability of a rapid OIT protocol using omalizumab in a phase 1 study to achieve desensitization to multiple (up to 5) food allergens in parallel, rapidly (7–36 weeks; median = 18 weeks). In the current long-term, observational study, we followed 34 food allergic participants for over 5 years, who had originally undergone the phase 1 rapid OIT protocol.

**Methods:**

After reaching the maintenance dose of 2 g protein for each of their respective food allergens as a part of the phase 1 study, the long-term maintenance dose was reduced for some participants based on a pragmatic team-based decision. Participants were followed up to 62 months through standard oral food challenges (OFCs), skin prick tests, and blood tests.

**Results:**

Each participant passed the 2 g OFC to each of their offending food allergens (up to 5 food allergens in total) at the end of the long-term follow-up (LTFU) study.

**Conclusion:**

Our data demonstrate the feasibility of long-term maintenance dosing of a food allergen without compromising the desensitized status conferred through rapid-OIT.

*Trial registration* Registry: Clinicaltrials.gov. Registration numbers: NCT01510626 (original study), NCT03234764 (LTFU study). Date of registration: November 29, 2011 (original study); July 26, 2017 (LTFU study, retrospectively registered)

**Electronic supplementary material:**

The online version of this article (10.1186/s13223-017-0223-8) contains supplementary material, which is available to authorized users.

## Background

Food Allergy is a serious immune response that disproportionately affects children [1–3] and its prevalence among children has increased over the past decade [4–7]. Food allergy is a burden for both the individual and the family, decreasing quality of life due to anxiety of potential reactions [8]. It can be associated with increased anxiety in the child [9].

There is no FDA approved therapy for food allergies, but oral immunotherapy (OIT), a treatment in which the patient eats small but slowly increasing doses of their allergen until they can tolerate a specified dose, was shown in research settings to be successful in children and adults for single foods as well as up to five foods in parallel [10–14].

In current care, individuals who are successfully desensitized to their offending food(s) at the end of OIT continue to ingest small amounts of these foods to maintain desensitization. However, studies following the participants after OIT long-term were focusing on single food OIT trials and without omalizumab as adjunct therapy [15–17].

In this observational, retrospective study, we followed the participants from a single site, phase 1 study in which omalizumab was used as adjunctive therapy with OIT (rapid-OIT) [14]. Our results for up to 62 months maximum during long-term follow-up (LTFU) dosing (after 2 g of food protein maintenance dose was reached for each food allergen by the participant) suggest that simultaneous desensitization to food allergens is possible over the long term.

## Methods

### Study design and participants

Participants who were successfully desensitized in a phase 1 omalizumab OIT (rapid-OIT) trial [14] [Investigational New Drug (IND) 14831, NCT01510626)] were invited to continue in an IRB-approved LTFU study at Stanford University. In the original publication [14], only a subset of participants with peanut in their OIT were included. However, additional participants with other food allergies (as determined by a validated food challenge) underwent the same protocol and were also invited to enroll in this LTFU study. The main additional allergies included allergies to almond, cashew, egg, hazelnut, milk, peanut, pecan, and walnut (Table 1). Quality of life studies for this cohort have been published [18, 19]. At the start of the LTFU study, the participants were switched from food flours/powders to food equivalents based on exact protein amounts for each specific food allergen. Participants of the LTFU study returned to the clinic on average every 6–12 months for questionnaires, skin tests, blood tests, and oral food challenges (OFCs). Initial maintenance dose in the phase 1 study was 2 g protein per food allergen. Then, for the LTFU, high (approximately 2 g protein) or low (approximately 300 mg protein) maintenance doses for each of the participant’s food allergens were selected in conjunction with the patient and the clinician. All participants were required to carry reaction medications throughout the LTFU study. Participants were closely monitored via routine clinic visits and phone calls during the LTFU study. Self-reported adverse Events were documented as per CTCAE v4.03 [20] criteria and according to regulatory guidelines. An independent Data Safety and Monitoring Board (DSMB) met every 6–12 months to review participant safety data.

**Table 1:** Demographics at the beginning of the phase I studies of participants who were enrolled in the LTFU study

Characteristics	LTFU participants
Number of ITT participants	34
Sex, male (n), female (n)	M (23), F (11)
Age at beginning of original phase I trial in years, median (range)	8.7 (4.6–16.9)
With other atopic conditions	
Atopic dermatitis (%)	57%
Atopic rhinitis (%)	73%
Asthma (%)	63%
With number food(s) in OIT	
1 food in OIT, n (%)	6 (17.6%)
2 foods in OIT, n (%)	7 (20.6%)
3 foods in OIT, n (%)	5 (14.7%)
4 foods in OIT, (n (%)	9 (26.5%)
5 foods in OIT, n (%)	7 (20.6%)
With food in OIT^a^	
Almond, n (%)	6 (17.6%)
Cashew, n (%)	18 (52.9%)
Egg, n (%)	11 (32.4%)
Hazelnut, n (%)	7 (20.6%)
Milk, n (%)	10 (29.4%)
Peanut, n (%)	26 (76.5%)
Pecan, n (%)	8 (23.5%)
Walnut, n (%)	10 (29.4%)

*OIT* oral immunotherapy

^a^Other foods with n = 1 not listed: e.g. Barely, Soy, Rye

### Oral food challenges, skin prick tests, and serological analysis

We conducted separate, standardized OFCs using validated and published techniques [21] with each food allergen that had been included in the OIT. For each allergen, OFCs were done in a staged approach with approximately 150, 300, 600, 1200, and 2000 mg of each food allergen on separate days. Skin prick tests (SPTs) were performed as per published methods [14].

Allergen-specific IgE and IgG_4_ levels were measured at Johns Hopkins Allergy and Clinical Immunology Reference Laboratory by ImmunoCAP fluorescence enzyme immunoassay (Thermo Fisher scientific/Phadia, Waltham, MA).

### Statistical analysis

Statistical analyses and preparation of graphics were performed in R. Kaplan–Meier methods were utilized to graphically depict time to reduction of long-term maintenance dose to for the separate foods. Differences between the Kaplan–Meier curves were assessed using a log-rank test (R package *survival* [22, 23]). The association of allergen specific IgG_4_ to IgE ratios (log 10) as well as SPT data with the two different dose levels (low vs. high maintenance dose) were evaluated for participants with available data using a linear mixed effects regression model controlling for the months of time point after maintenance was reached with random effects for participant and allergen type. Similarly, we fitted a linear mixed effects regression model to assess the association of the outcomes of the OFCs to 2 g throughout the LTFU study with the two different dose levels (low vs. high maintenance dose), controlling for the months of time point after maintenance was reached, the allergen type and the participant.

## Results

### Study participants and characterization of their food allergies

**Fig. 1:**
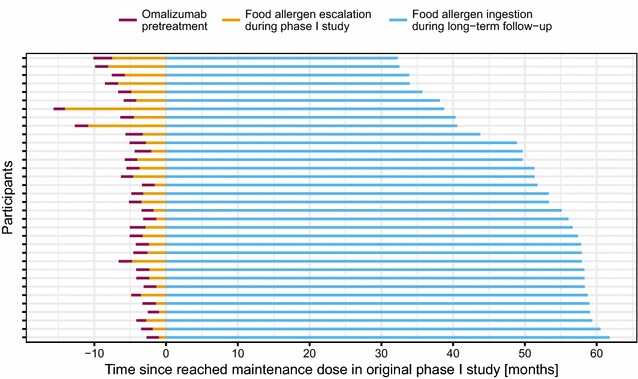
Timeline of original phase 1 trial and long-term follow-up study. Zero depicts the time at which the participant reached their 2 g maintenance dose during the original phase 1 trial

**Fig. 2:**
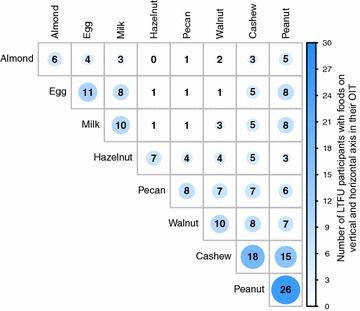
Number of foods in participant’s OIT. The diagonal shows the number of participants, who ingest maintenance doses of the named food. The other numbers show the number of participants with the two foods on the vertical and horizontal axis in their OIT. This is independent of other possible additional foods in the OIT. A greater number is also reflected by a darker color and a greater circle

In this LTFU study, 34 participants previously desensitized to 1 or up to 5 foods in parallel during a rapid-OIT trial (with 16 weeks of adjunct omalizumab), were followed. Methods for the rapid desensitization are published elsewhere [14]. The participants were followed for up to 62 months after the respective participant reached the 2 g maintenance dose for the first food (median 53 months, range 32–62 months, Fig. 1). The participants entered the LTFU study at different dates and they were followed until May 2017, which resulted in the variation of their time in the LTFU study. A summary of the clinical characteristics and demographics of the participants who enrolled in the LTFU study is displayed in Table 1. The largest proportion of participants (n = 9, 26.5%) was dispensed four food allergens in their OIT. The most common food allergen was peanut (n = 26, 76.5%) and the second most common food allergen in the OIT was cashew with 18 (52.9%) participants. Overall, we followed the long-term maintenance dosing of eight different food allergens (Table 1). The participants were desensitized to various combinations of these food allergens. It was previously shown in a study of DBPCFCs in 60 multi-allergic children that several combinations of food allergies often occur together [24]. The number of participants that were sensitized, underwent OIT and then followed for each pairwise combination of two foods is shown in Fig. 2. Of the 11 participants that were desensitized to egg and the 10 that were desensitized to milk, 8 were desensitized to both of these foods. Of the 8 participants with pecan in their OIT and 10 with walnut, 7 were desensitized to both foods, corresponding with previous reports of cosensitization between pecan and walnut [25, 26].

### Long-term maintenance dosing and OFC

We followed the 34 participants for a maximum of 62 months after they reached their 2 g protein maintenance dose for the first food allergen in their OIT, total of up to 65 months of dosing. Throughout the follow-up phase for each participant for each food allergen the decision to stay on a high LTFU maintenance dose per food allergen or reduce to a low LTFU maintenance dose per food allergen was made between the clinician and the parent in a collaborative approach based on adherence and taste. No change of dose occurred because of safety reasons and each dose change was monitored closely by the trained clinical team.

**Fig. 3:**
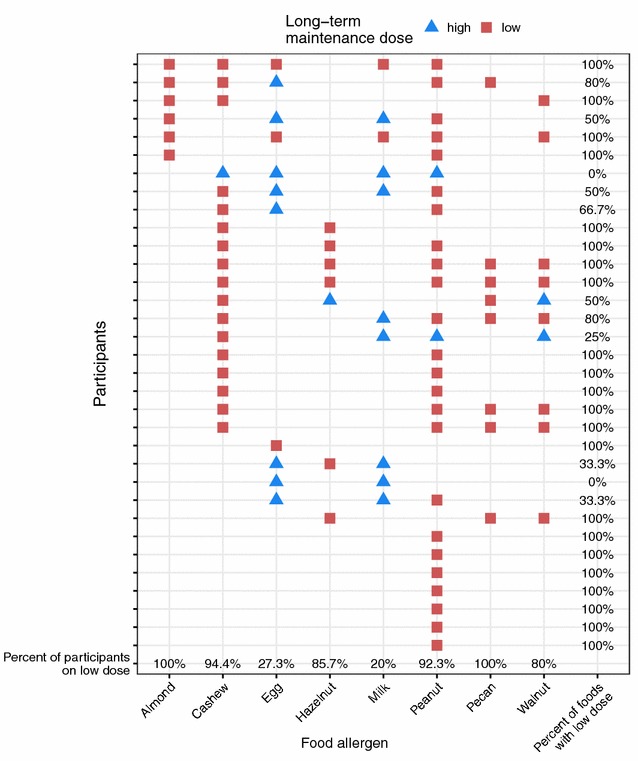
Long-term maintenance doses at the end of the LTFU study. Each row shows one of the 34 participants. The red squares and the blue triangles depict that the food allergen in that column is ingested at a low long-term or a high long-term maintenance dose, respectively. The last row shows the percentage of participants that were desensitized to that food and ingesting it at a low maintenance dose at the conclusion of the LTFU. The last column shows the percentage of the food allergens per participant that the participant ingests at a low long-term maintenance dose

The long-term maintenance doses at the end of our follow-up phase in May 2017 for each participant for each food are shown in Fig. 3. All doses shown as “low” were 300 mg of food and the “high” doses ranged from 2 to 4 g. All six participants that were desensitized to almond and all eight participants that had pecan in their OIT ate these foods on a low maintenance dose at the end of the follow-up phase. On the other hand, only 27% (3 participants) of the participants desensitized to egg and only 20% (2 participants) desensitized to milk were on a low long-term maintenance dose for these foods. Our study found that egg and milk were the two allergens that were consumed most often at a high long-term maintenance dose (Fig. 3). This most probably is due to the fact that many foods contain egg and milk at high protein levels. Only two participants stayed on a high maintenance dose throughout the LTFU study for all their foods (2 and 4 foods in OIT, respectively).

Each participant performed separate OFCs at the end of our follow-up phase. In these challenges, every person could tolerate at least 2 g of each of their food allergens without symptoms in the OFC, independent of a low or high long-term maintenance dose.

The outcome of 2 g OFCs throughout the course of the LTFU study was not significantly associated with the participant being on a low or high maintenance dose for the allergen at that time point (P = 0.69). However, this result has to be interpreted with care, considering the small sample size and the small percentage of failed OFCs (3.5% of all 464 performed OFCs).

### Timeline of maintenance dosing

**Fig. 4:**
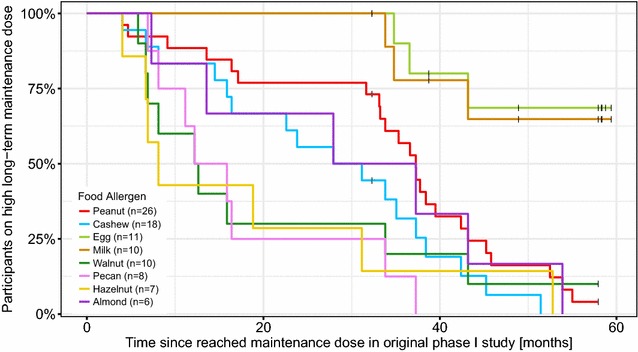
Kaplan-Meier curves of participants on a high maintenance dose over time stratified by allergen. The percentage of participants per allergen continuing on a high long-term maintenance dose is shown over time. Black lines show the latest visits of participants continuing on a high long-term maintenance dose per allergen at the end of our follow-up

The changes from the high to a low LTFU maintenance dose occurred at different times past the initial maintenance dose of 2 g for each food allergen in the OIT per each individual in the study. The Kaplan–Meier plot in Fig. 4 shows the percentage of participants who continued the high long-term maintenance dose per food (as opposed to low long-term maintenance dose) over time. Two participants increased their low maintenance dose again to a high dose during our follow-up phase (not shown in the Kaplan–Meier plot). One of these participants was on a low dose for all five foods, including milk for which the dose was changed after 12 months from a low to a high dose due to the individual wanting to eat more dairy products. This change was done under supervision of our clinicians and it was not due to safety issues with milk but because of preferences of the child and parent. The same applied to the other participant who increased the dose for walnut and hazelnut (out of four foods in total in OIT) from a low to a high maintenance dose after 39 months on the low dose and this increase occurred without problems. Both participants had no allergic symptoms after the increase of their doses.

### Clinical outcomes: serology and SPTs

For several participants, we measured their allergen-specific IgE and IgG_4_ at sequential time points during LTFU. The data for the IgG_4_/IgE ratios during the phase 1 trial dose escalation and subsequent time points are shown in Additional file 1: Figure S1. The log10 IgG_4_/IgE ratio levels were not significantly different between a low and a high maintenance dose (P = 0.34) after adjusting the data for the months after the 2 g maintenance dose was reached, the participant, and the type of food allergen.

The SPT data show that the wheal diameter decreased during the dose escalation phase (Additional file 2: Figure S2). During the LTFU phase, the wheal diameter varied across a large range for the participants per allergen, however, it was not significantly dependent on a low or high maintenance dose (P = 0.1).

### Safety

The number of allergic reactions during the LTFU study decreased over time (Table 2). In total 1126 reactions were recorded (3.5% of maintenance doses). Of those, 1076 reactions (95.6%) were mild, 40 (3.6%) were moderate and 5 (0.4%) were classified as severe. There were no fatal events. There were no serious adverse events. There were no anaphylactic reactions. Four of the severe adverse events were skin reactions and one was nasal congestion. All five severe reactions occurred within the first 19 months after participants reached the maintenance dose and while each participant was on a 2 g maintenance dose for their food allergens. No severe respiratory, gastrointestinal or hypotension adverse events occurred. Epinephrine was used by participants for mild or moderate reactions associated with minor wheezing. There were no events involving shortness of breath, dyspnea, hypotension, dizziness, or difficulty breathing. Safety results did not differ based on low vs high LTFU maintenance dose.

**Table 2:** Reaction rates associated with allergic reactions after dosing by organ system, grade, and time period of LTFU study

Reaction numbers and % of total reactions	Reaction numbers for ITT	Month 0–6	Month 7–19	Month 20–32	Month 33–62
Number of participants		34	34	34	32
Total ITT reactions	1126	671	342	99	14
Gastrointestinal	71 (6.3%)	47 (7%)	22 (6.4%)	2 (2%)	0 (0%)
Mild	71	47	22	2	0
Moderate	0	0	0	0	0
Severe	0	0	0	0	0
Respiratory/thoracic/mediastinal	55 (4.9%)	39 (5.8%)	16 (4.7%)	0 (0%)	0 (0%)
Mild	52	37	15	0	0
Moderate	3	2	1	0	0
Severe	0	0	0	0	0
Skin/subcutaneous	569 (50.5%)	302 (45%)	202 (59.1%)	61 (61.6%)	4 (28.6)
Mild	530	270	196	60	4
Moderate	35	30	4	1	0
Severe	4	2	2	0	0
Eye	25 (2.2%)	21 (3.1%)	2 (0.6%)	2 (2%)	0 (0%)
Mild	25	21	2	2	0
Moderate	0	0	0	0	0
Severe	0	0	0	0	0
Cardiovascular	0 (0%)	0 (0%)	0 (0%)	0 (0%)	0 (0%)
Nasal congestion	227 (20.2%)	128 (19.1%)	71 (20.8%)	21 (21.2%)	7 (50%)
Mild	219	120	71	21	7
Moderate	7	7	0	0	0
Severe	1	1	0	0	0
Other (i.e. anxiety)	179 (15.9%)	134 (20%)	29 (8.5%)	13 (13.1%)	3 (21.4%)
Mild	179	134	29	13	3
Moderate	0	0	0	0	0
Severe	0	0	0	0	0

The number of participants that dosed during each months period is given as n

## Discussion

The efficacy of OIT for food allergy to desensitize to one or several foods has been demonstrated in several trials [10–14, 27, 28]. It is important to study the follow-up of participants after they finished the desensitization. Published follow-up studies [15–17, 29, 30] mostly focus on participants who continue on or increase their achieved maintenance dose and who were desensitized to only a single food mostly without omalizumab therapy. In Meglio et al. [17], families who participated in a OIT trial for cow’s milk were evaluated by a structured interview after an average 4 years and 8 months after start of their OIT. The parents were advised at the end of the original trial not to discontinue the daily intake of cow’s milk (with the goal of maintaining the effects of the oral desensitization) and the follow-up results indicated a persistence of the desensitization effect [17]. Jones et al. [15] followed participants of an egg OIT trial for up to 4 years in which the participants continued egg intake or (for a shorter period) placebo. At the end of the study, the participants were tested for sustained unresponsiveness, which 50% of the egg-consuming group achieved. In a recent blinded, phase 2 clinical trial, it was shown that OIT in combination with omalizumab enables rapid and safe desensitization in multifood allergic participants. Desensitization was achieved more successfully with omalizumab than placebo as an adjunctive therapy with OIT over 36 weeks in this trial [31]. In this current observational study, we addressed a different question. Can the maintenance dose be reduced after maintenance is achieved in OIT and still maintain the effect of desensitization? Many children have aversions to the high maintenance dose of each food allergen due to taste, convenience, sense of fullness, etc. A lower maintenance dose might increase not only an individual’s quality of life but also an individual’s adherence with continued ingestion of allergens long-term.

Here we present an observational, retrospective study of the LTFU of children after OIT with omalizumab as adjunctive therapy (omalizumab used for the first 16 weeks in the phase 1 study). The 34 participants were followed for a maximum of 62 months after they reached the 2 g maintenance dose in the original phase I trial [14], total up to 65 months after starting OIT. Throughout this time, the participants returned to the clinic on average every 6–12 months and OFCs up to 2 g for all offending foods were performed by a board-certified allergy and immunology specialist. All participants started the LTFU study at a maintenance dose of at least 2 g protein per food allergen and they either continued on this dose or reduced their dose to a low (median 300 mg) dose. This was not a randomized, placebo-controlled study but the long-term maintenance doses were decided throughout the study by the clinicians in collaboration with the parents of the participating children. Our reason for patient-based participation with a clinician was that this reflected more of a ‘real-life scenario’ and the decision to reduce the dose was not because of safety reasons but because of preference and ease of dosing compliance.

At the end of the LTFU study, of all 96 food allergens across the 34 participants, 74 (77%) were taken at a low, long-term maintenance dose (Fig. 3). Every participant passed the 2 g OFC to each of their offending foods at the end of the follow-up phase in May 2017. No severe respiratory, gastrointestinal or hypotension reactions occurred after dosing and the overall rate of adverse events decreased over the course of the study.

This study has several strengths. The follow-up was done by in-person visits, including questionnaires but also standardized OFCs were performed to quantify the desensitization status for each food allergen. The study also has several limitations. The number of participants with each individual food and food allergen combination is too low to give adequate power for statistical analyses of these groups. Furthermore, the IgG_4_/IgE ratio or SPT wheal diameter data was limited in the study by some of the participants during LTFU (i.e. a convenience sample based on those individuals who agreed to blood and/or skin tests).

## Conclusion

Our LTFU cohort of food OIT individuals who received omalizumab for the first 16 weeks during the original phase 1 trial supports the feasibility of long-term dosing in food-allergic individuals.

## Additional files

**Additional file 1: Figure S1.** Allergen-specific IgG_4_/IgE ratios for various time points during the dose escalation and after 2 g maintenance dose was reached. Each line represents one participant. The dots are colored by the dose at the specific time point.

**Additional file 2: Figure S2.** Wheal diameter of SPTs for various time points during the dose escalation and after 2 g maintenance dose was reached. Each line represents one participant. The dots are colored by the dose at the specific time point.

## Abbreviations

LTFU: long-term follow-up
OIT: oral immunotherapy
OFC: oral food challenge
SPT: skin prick test

## References

[1] Gupta RS, Springston EE, Warrier MR, Smith B, Kumar R, Pongracic J (2011). The prevalence, severity, and distribution of childhood food allergy in the United States. Pediatrics.

[2] Liu AH, Jaramillo R, Sicherer SH, Wood RA, Bock SA, Burks AW (2010). National prevalence and risk factors for food allergy and relationship to asthma: results from the National Health and Nutrition Examination Survey 2005–2006. J Allergy Clin Immunol.

[3] Sampson HA (2004). Update on food allergy. J Allergy Clin Immunol.

[4] Branum AM, Lukacs SL (2008). Food allergy among US children: trends in prevalence and hospitalizations.

[5] Decker WW, Campbell RL, Manivannan V, Luke A, St Sauver JL, Weaver A (2008). The etiology and incidence of anaphylaxis in Rochester, Minnesota: a report from the Rochester Epidemiology Project. J Allergy Clin Immunol..

[6] Grundy J, Matthews S, Bateman B, Dean T, Arshad SH (2002). Rising prevalence of allergy to peanut in children: data from 2 sequential cohorts. J Allergy Clin Immunol.

[7] Jackson KD, Howie LD, Akinbami LJ (2013). Trends in allergic conditions among children: United States, 1997–2011.

[8] Gupta RS, Kim JS, Barnathan JA, Amsden LB, Tummala LS, Holl JL (2008). Food allergy knowledge, attitudes and beliefs: focus groups of parents, physicians and the general public. BMC Pediatr.

[9] Flokstra-de Blok BM, Dubois AE, Vlieg-Boerstra BJ, Oude Elberink JN, Raat H, DunnGalvin A (2010). Health-related quality of life of food allergic patients: comparison with the general population and other diseases. Allergy.

[10] Jones SM, Burks AW, Dupont C (2014). State of the art on food allergen immunotherapy: oral, sublingual, and epicutaneous. J Allergy Clin Immunol.

[11] Burks AW, Jones SM, Wood RA, Fleischer DM, Sicherer SH, Lindblad RW (2012). Oral immunotherapy for treatment of egg allergy in children. N Engl J Med.

[12] Blumchen K, Ulbricht H, Staden U, Dobberstein K, Beschorner J, de Oliveira LC (2010). Oral peanut immunotherapy in children with peanut anaphylaxis. J Allergy Clin Immunol.

[13] Begin P, Winterroth LC, Dominguez T, Wilson SP, Bacal L, Mehrotra A (2014). Safety and feasibility of oral immunotherapy to multiple allergens for food allergy. Allergy Asthma Clin Immunol.

[14] Begin P, Dominguez T, Wilson SP, Bacal L, Mehrotra A, Kausch B (2014). Phase 1 results of safety and tolerability in a rush oral immunotherapy protocol to multiple foods using Omalizumab. Allergy Asthma Clin Immunol.

[15] Jones SM, Burks AW, Keet C, Vickery BP, Scurlock AM, Wood RA (2016). Long-term treatment with egg oral immunotherapy enhances sustained unresponsiveness that persists after cessation of therapy. J Allergy Clin Immunol.

[16] Keet CA, Seopaul S, Knorr S, Narisety S, Skripak J, Wood RA (2013). Long-term follow-up of oral immunotherapy for cow’s milk allergy. J Allergy Clin Immunol.

[17] Meglio P, Giampietro PG, Gianni S, Galli E (2008). Oral desensitization in children with immunoglobulin E-mediated cow’s milk allergy–follow-up at 4 year and 8 months. Pediatr Allergy Immunol.

[18] Arasi S, Otani IM, Klingbeil E, Begin P, Kearney C, Dominguez TL (2014). Two year effects of food allergen immunotherapy on quality of life in caregivers of children with food allergies. Allergy Asthma Clin Immunol.

[19] Otani IM, Begin P, Kearney C, Dominguez TL, Mehrotra A, Bacal LR (2014). Multiple-allergen oral immunotherapy improves quality of life in caregivers of food-allergic pediatric subjects. Allergy Asthma Clin Immunol.

[20] National Institute of Cancer. Common terminology criteria for adverse events (CTCAE).v4. 2009. https://evs.nci.nih.gov/ftp1/CTCAE/CTCAE_4.03_2010-06-14_QuickReference_5x7.pdf. Accessed 8 Aug 2017.

[21] Sampson HA, Gerth van Wijk R, Bindslev-Jensen C, Sicherer S, Teuber SS, Burks AW (2012). Standardizing double-blind, placebo-controlled oral food challenges: American Academy of Allergy, Asthma and Immunology-European Academy of Allergy and Clinical Immunology PRACTALL consensus report. J Allergy Clin Immunol.

[22] Therneau TM. A package for survival analysis in S. v 2.41.3. 2015. https://cran.r-project.org/web/packages/survival/index.html. Accessed 2 Aug 2017.

[23] Therneau TM, Grambsch PM (2000). Modeling survival data: extending the Cox model.

[24] Andorf S, Borres MP, Block W, Tupa D, Bollyky JB, Sampath V (2017). Association of clinical reactivity with sensitization to allergen components in multifood-allergic children. J Allergy Clin Immunol Pract.

[25] Goetz DW, Whisman BA, Goetz AD (2005). Cross-reactivity among edible nuts: double immunodiffusion, crossed immunoelectrophoresis, and human specific igE serologic surveys. Ann Allergy Asthma Immunol.

[26] Uotila R, Kukkonen AK, Pelkonen AS, Makela MJ (2016). Cross-sensitization profiles of edible nuts in a birch-endemic area. Allergy.

[27] Jones SM, Pons L, Roberts JL, Scurlock AM, Perry TT, Kulis M (2009). Clinical efficacy and immune regulation with peanut oral immunotherapy. J Allergy Clin Immunol.

[28] Skripak JM, Nash SD, Rowley H, Brereton NH, Oh S, Hamilton RG (2008). A randomized, double-blind, placebo-controlled study of milk oral immunotherapy for cow’s milk allergy. J Allergy Clin Immunol..

[29] Burks AW, Wood RA, Jones SM, Sicherer SH, Fleischer DM, Scurlock AM (2015). Sublingual immunotherapy for peanut allergy: long-term follow-up of a randomized multicenter trial. J Allergy Clin Immunol.

[30] Narisety SD, Skripak JM, Steele P, Hamilton RG, Matsui EC, Burks AW (2009). Open-label maintenance after milk oral immunotherapy for IgE-mediated cow’s milk allergy. J Allergy Clin Immunol.

[31] Andorf S, Purington N, Block WM, Long AJ, Tupa D, Brittain E, Spergel AR, Desai M, Galli SJ, Nadeau KC, Chinthrajah RS (2017). Anti-IgE treatment with oral immunotherapy in multifood allergic participants: a double-blind, randomised, controlled trial. Lancet Gastroenterol Hepatol.

