# Supplementary material for: Observational long-term follow-up study of rapid food oral immunotherapy with omalizumab
Source: Allergy Asthma Clin Immunol. 2017 Dec 21;13:51. doi: 10.1186/s13223-017-0223-8 (PMC5738812; doi:10.1186/s13223-017-0223-8)
Supplement: Supplementary file 1 — Additional file 1: Figure S1. Allergen-specific IgG4/IgE ratios for various time points during the dose escalation and after 2 g maintenance dose was reached. Each line represents one participant. The dots are colored by the dose at the specific time point. [file 13223_2017_223_MOESM1_ESM.docx]

Supplementary Figure S1: Allergen-specific IgG_4_/IgE ratios for various time points during the dose escalation and after 2 g maintenance dose was reached. Each line represents one participant. The dots are colored by the dose at the specific time point.
